# Patient-specific biophysical AI-powered modeling for predicting RFA volumes in HCC

**DOI:** 10.1186/s41747-026-00839-3

**Published:** 2026-11-03

**Authors:** Chloé Audigier, Fouad Georges Akkari, Felix Meister, Andrea Tonglet, Bénédicte Maréchal, Oliver Frings, Rafael Duran

**Affiliations:** 1 Siemens Healthineers, Swiss Innovation Hub, Lausanne, Switzerland; 2 Department of Radiology and Interventional Radiology, Lausanne University Hospital, Lausanne, Switzerland; 3 Siemens Healthineers, Digital Technologies and Innovation, Erlangen, Germany

**Keywords:** Carcinoma (hepatocellular), Computer simulation, Deep learning, Planning (computer-assisted), Radiofrequency ablation

## Abstract

**Objectives:**

To clinically evaluate a fully automated, artificial intelligence-powered, patient-specific biophysical model for predicting radiofrequency ablation (RFA) volumes in hepatocellular carcinoma (HCC) and to compare its performance with manufacturer-provided ablation estimates.

**Materials and methods:**

This retrospective study included 96 RFA procedures in 65 HCC patients treated using a single ablation with a cluster needle. Tumors, needle position, and postablation zone were manually segmented on periprocedural computed tomography. Liver parenchyma and vascular structures were automatically extracted using deep learning. A graphics processing unit (GPU)-accelerated biophysical model simulated heat transfer, vascular heat-sink effects, and tissue necrosis using patient-specific anatomy and procedural data. Correlations between ablation volume manually segmented and clinical or anatomical parameters (ablation time, ablation tip temperature, tumor–capsule distance, vessel proximity, type, and diameter) were assessed. Model-predicted volumes were compared with ground truth and manufacturer estimates using relative volume difference (RVD) and volume similarity.

**Results:**

Final ablation volumes showed wide variability (7.9–78.8 cm³) and were not correlated with any individual clinical or anatomical parameter (Pearson *r* – 0.02 to 0.29). Across 94 evaluable cases, simulated and ground-truth volumes were similar (*p* = 0.165), while manufacturer estimates significantly overestimated ablation volume (*p* < 0.001). The model achieved a median RVD of 0.39 (interquartile range 0.24–0.57), significantly lower than the manufacturer's algorithm (1.04; 0.64–1.61). Incorporating patient-specific ablation time further improved accuracy. Simulation was fast (0.45 s per second of ablation on GPU).

**Conclusion:**

A fully automated, patient-specific biophysical model substantially improves RFA ablation volume prediction in HCC compared with manufacturer approximations, supporting its potential role in personalized treatment planning and outcome standardization.

**Key Points:**

***Question*** In patients with hepatocellular carcinoma, can a fully automated, AI-powered, patient-specific biophysical model predict radiofrequency ablation volume more accurately than manufacturer-provided estimates?

***Findings*** A fully automated, AI-powered biophysical model integrating patient-specific anatomy and vasculature predicted RFA volume significantly better than manufacturer estimates (median relative volume difference (RVD) 0.39 *versus* 1.04).

***Relevance statement*** Accurate ablation volume prediction could support personalized radiofrequency ablation (RFA) planning, reduce reliance on operator experience, and standardize treatment margins in hepatocellular carcinoma (HCC), potentially improving local tumor control and reducing recurrence in cirrhotic patients undergoing thermal ablation.

**Graphical Abstract:**

**Figure:**
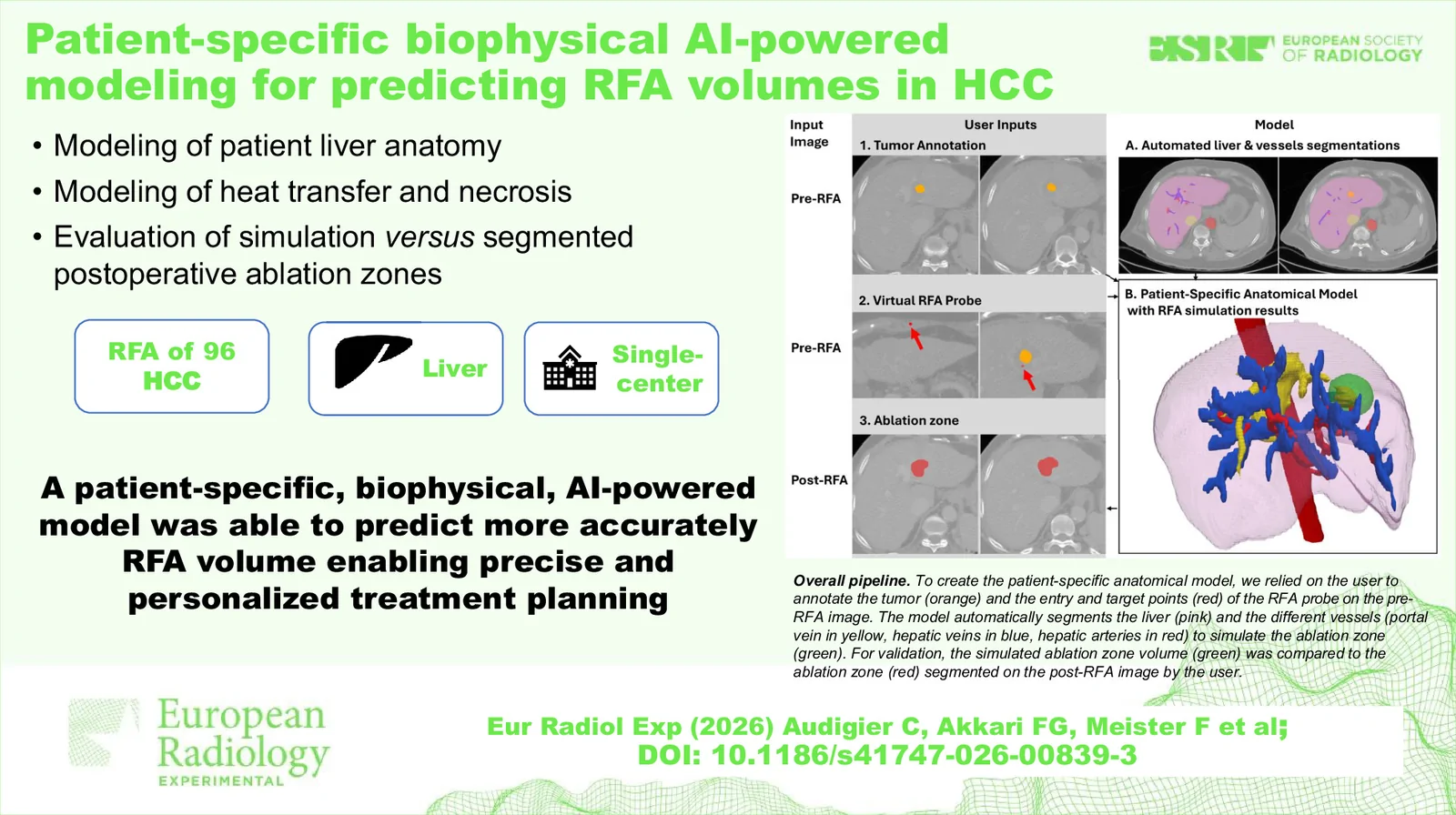

## Introduction

Radiofrequency ablation (RFA) is an established, minimally invasive technique for treating hepatic malignancies [1, 2]. The procedure involves image-guided percutaneous needle insertion into the tumors to achieve heat-induced tissue necrosis. Successful ablation requires tumor safety margins of at least 5 mm to avoid tumor residue or recurrence [3–5], which highly depends on the operator’s experience at choosing the type and number of needles, their placement, and the appropriate ablation settings (*i.e*., ablation power and duration) [6].

However, predicting the final ablation volume to ensure sufficient margins remains challenging. RFA needle manufacturers provide expected ablation zones for specific ablation settings, but they are typically spherical or ellipsoidal approximations derived from idealized *ex vivo* data. Several patient-specific factors, such as liver parenchyma condition (*e.g*., fibrosis, cirrhotic tissue) [7, 8], blood perfusion, and proximity to large vessels [9, 10], significantly influence the ablation outcome. The heat-sink effect [11, 12], particularly relevant if large vessels (diameter > 5 mm) are close to the ablation zone, can reduce the RFA efficacy and cause local recurrence [13, 14].

Consequently, effective planning is essential for improving ablation outcomes. Computational modeling is a promising approach for predicting the ablation zone by simulating heat diffusion and incorporating patient-specific parameters, such as vascular anatomy and hepatic perfusion [15–18]. These models enable virtual testing of different ablation strategies before the actual procedure [19], supporting optimal needle placement and treatment planning. While some approaches have been evaluated in the context of microwave ablation and RFA, studies remain limited in scale [20, 21] and often require manual interventions [22, 23]. Although distinctions between HCC and other hepatic tumors are often overlooked [22], it is crucial to consider the liver background, as HCC typically arises in fibrotic or cirrhotic livers characterized by distinct thermal properties.

In this paper, we present a retrospective clinical evaluation of HCC patients using a fully automated, artificial intelligence-powered, patient-specific biophysical model that simulates the physical mechanisms underlying RFA. Our approach introduces patient-specificity by integrating individual liver anatomy, vascular structures, and treatment parameters, which are automatically extracted from intra-procedure computed tomography (CT) images and ablation durations.

We aimed to: (1) investigate whether manually measured, patient-specific parameters such as the distance between the tumor and liver capsule, the distance between the tumor and the closest vessel, the type and diameter of the closest vessel, the ablation tip temperature measured intra-operatively at the end of the ablation protocol, and the ablation time could predict the ablation volume; (2) demonstrate the feasibility of a fully automated, AI-powered biophysics-based simulation for RFA prediction; (3) evaluate the model’s predictive power in a large-scale HCC patient cohort; and (4) compare the model predictions with the probe manufacturer’s specifications.

## Materials and methods

This retrospective study of a prospectively collected patient cohort (2011–2022) was approved by the Cantonal research ethics committee for research on humans, Vaud (CER-VD), Switzerland (CER-VD: 2023-00640). Informed consent was waived. The study complied with the principles of the Declaration of Helsinki.

### Patient selection

Inclusion criteria were: (1) biopsy-proven or radiologically confirmed HCC; (2) HCC treated with RFA only (up to 3 cm); (3) RFA performed using one probe; (4) treatment consisting of a single impact (3 roll-offs) per treated tumor without needle repositioning; and (5) an imaging dataset composed of multiphasic contrast-enhanced CT immediately before, during, and after RFA. Exclusion criteria were: (1) HCC lesion, final needle position and/or final ablation zone not visible in the CT images; and (2) presence of relevant image artefacts.

### Ablation protocol

All patients were presented at a multidisciplinary liver tumor board and gave consent for the procedure. Patients underwent RFA under general anesthesia using jet ventilation. CT scans were performed with arms placed above the head using a dedicated interventional CT suite (Philips Ingenuit) or Alphenix 4DCT (Canon Medical Systems Corporation). Once the patient was anesthetized on the treatment table, a non-enhanced CT followed by a contrast-enhanced CT in the arterial and portal venous phases was performed.

CT images were used to localize the tumor and to plan treatment. The RFA needle was percutaneously positioned into the targeted tumor by the interventional radiologist using ultrasound or CT guidance. If needed, intermittent imaging was acquired until adequate needle positioning was obtained. A non-enhanced CT was acquired to verify the needle placement before ablation. No guidance software was used.

The probe used was the Cool-Tip^TM^ RF Ablation Cluster Electrode (Medtronic), with an exposure of 2.5 cm and a length of 10, 15, or 20 cm (depending on the tumor depth and patient morphology), with an ablation protocol initially set at 12 min. As the RF needle is impedance-controlled, a roll-off occurs when the system detects a significant increase in impedance, causing the generator to automatically shut off. 3 roll-offs were systematically used as the ablation endpoint, without enforcing the 12-min time limit. This indicates that the desired ablation has been theoretically obtained according to the manufacturer’s specifications and regardless of the lesion dimensions: an ellipsoid with a long axis length of *A* = 45 mm, and a short axis length of *B* = 42 mm. After the ablation was finished, the RFA probe was removed, and a multiphasic contrast-enhanced CT scan was performed to evaluate the ablation zone and to identify potential treatment-related complications.

### Image annotation and anatomical measurements

Manual segmentations and measurements were carried out by two board-certified radiologists, each with over 3 years of experience. The needle position, tumor, and ablation zone segmentations were manually outlined on the per-procedure multiphasic contrast-enhanced CT scans using ITK-SNAP [24]. Tumor segmentation was based on the pretreatment acquisition, in which it was most clearly visible, typically in the arterial phase, and was performed slice by slice on axial slices, then verified and corrected in coronal and sagittal planes to ensure volumetric accuracy and spatial consistency. The ablation zone was segmented in the same manner from the post-treatment venous-phase acquisition. For the needle position, annotations were made on the pre-treatment venous-phase acquisition, referencing the CT scan acquired when the needle was in its final position. Two specific points were marked: the distal tip of the needle and the point where the needle intersects the skin. This approach allowed for an accurate representation of the needle trajectory relative to the tumor without requiring image registration.

We also measured characteristics of HCC localization and its environment: distance to the hepatic capsule, type of the closest vessel (portal vein, hepatic vein or hepatic artery), diameter of the closest vessel at its closest point and its distance to the border of the tumor. The closest vessel to the tumor was determined using the pre-procedure imaging, in which it was best visible. All measurements were carried out on axial slices, considering only vessels located within 15 mm of the tumor border.

### Fully automated patient-specific thermal ablation modeling

To simulate heat transfer within hepatic tissue, we used a biophysics-based model composed of the four main components described below.

#### Model of patient liver anatomy

**Fig. 1:**
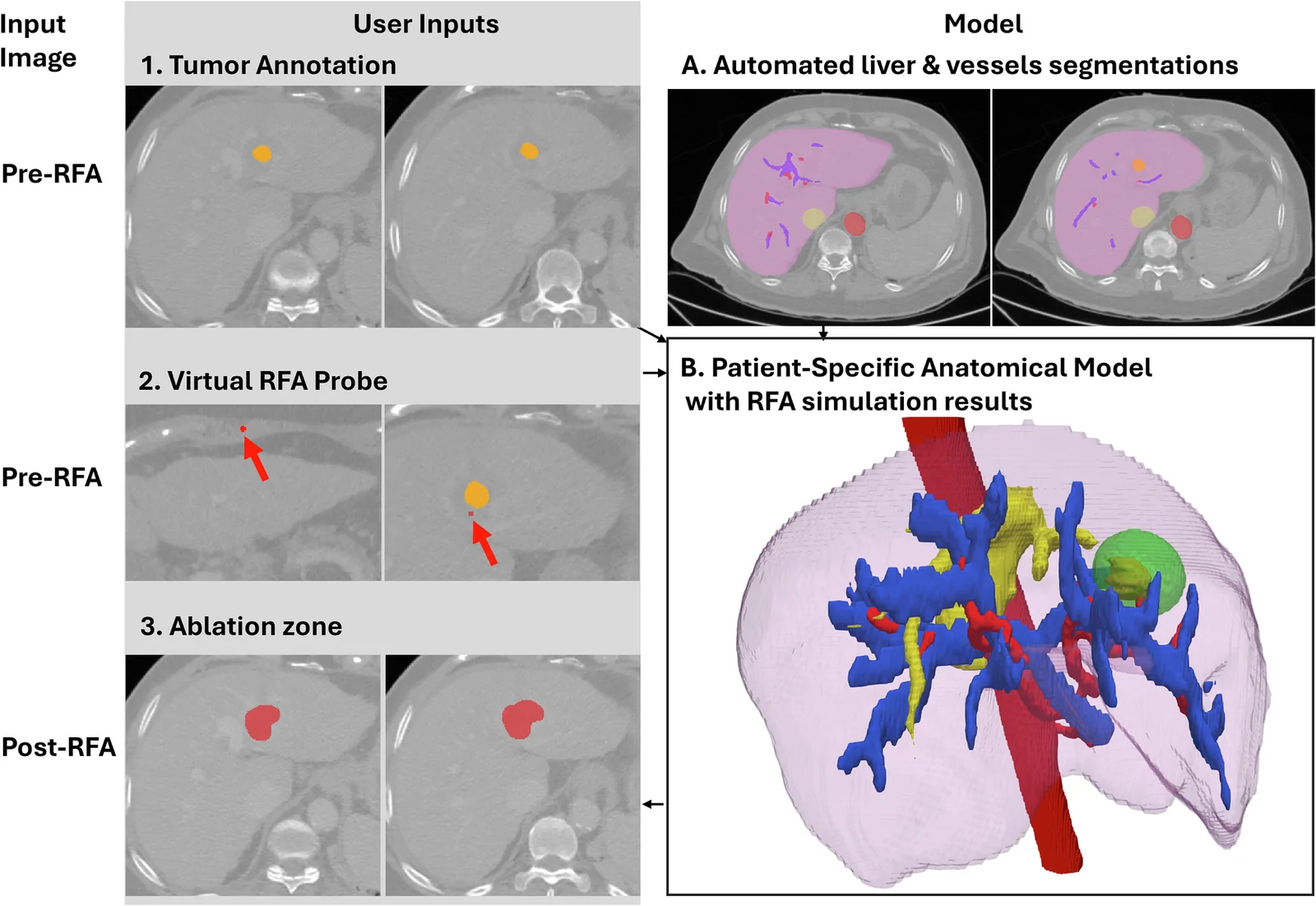
Overall pipeline. To create the patient-specific anatomical model, we relied on the user to annotate the tumor (orange) and the entry and target points (red) of the RFA probe on the pre-RFA image. The model automatically segments the liver (pink) and the different vessels (portal vein in yellow, hepatic veins in blue, hepatic arteries in red) to simulate the ablation zone (green). For validation, the simulated ablation zone volume (green) was compared to the ablation zone (red) segmented on the post-RFA image by the user. RFA, Radiofrequency ablation

We automatically segmented the liver parenchyma, portal veins, hepatic veins and arteries using a deep learning image-to-image network [25], based on the phase of the pre-RFA contrast-enhanced CT image in which the tumor was annotated. The overall pipeline for the Patient-Specific Anatomical model and the resulting automated segmentations are illustrated in Fig. 1.

#### Model of heat transfer in hepatic tissue

Consistent with other thermal ablation simulation software [15–23], our biophysics model is derived from [26]. Specifically, we used the bioheat equation (Eq. 1) to simulate the temperature propagation from the RF needle through the liver while considering the cooling effect of the major blood vessels:

1 $$\rho c\,\partial T/\partial t=Q+\nabla \cdot (d(T)\nabla T)+{I}_{V}\omega ({T}_{{{{\rm{blood}}}}}-T)$$

where $\rho ,{c}$, *d(T)*, and ***ω*** are the density, specific heat capacity, temperature-dependent thermal conductivity of tissues and heat transfer coefficient, respectively. *t* is the time, *Q* the heat source term, *T* and *T*_blood_ represent the tissue and blood temperature, which is assumed to be constant in large vessels. ${{{{\boldsymbol{I}}}}}_{{{{\boldsymbol{V}}}}}$ is the vessel indicator function, allowing the reaction term to be applied only in the segmented hepatic blood vessels. We used a Neumann boundary condition at the liver border.

#### Model of necrosis

A three-state model [27] estimates the necrosis zone by computing the concentration of alive (A), vulnerable (V), and dead (D) cells over time based on the simulated temperature.

The model assumes that cells stop functioning if the temperature exceeds a certain threshold for a sufficient duration. If this threshold is not maintained long enough, the affected cell will recover. This model is fully coupled with the heat transfer model since the heat capacity is updated based on the cell state [16].

#### Model of a heat source

We used a Dirichlet boundary condition to model the source term *Q*. The points inside an ellipsoid of fixed axis A_source_ and B_source_ mm centered at the RF needle tip, with a distal throw (distance beyond the probe’s tip where the ablation extends) D_source_, are enforced at temperature *T*_source_ for a duration of *t*_abl_ seconds followed by *t*_2_ seconds without the Dirichlet boundary condition to reach a steady state. We chose an ellipsoidal heat source because the manufacturer-reported expected ablation zone is derived from *ex vivo* data and, in this diffusion-dominated setting, the imposed source geometry largely determines the predicted ablation shape.

For implementation, we used a Lattice Boltzmann Method for fast computation on a GPU to solve Eq. 1 with a timestep of *d*_*t*_ and a spatial resolution of *d*_*x*_*;* and simulated the temperature and necrosis distributions over time. The same tissue parameters, listed in Table 1, were used for all patients. Technical and clinical parameters were chosen from [16] or according to the manufacturer’s specifications.

**Table 1:** Parameters of the computational model: definition, units, and values used in the present study

Parameter	Description	Value
*d*_x_	Spatial resolution	1 mm
*d*_t_	Time resolution	0.1 s
*t*_abl_	Ablation duration	Patient-specific
*t*_2_	Cooling duration	180 s
*d*	Blood and tissue heat conductivities	0.512 ° (1 + 0.00161 × (T − 37)) W (m K) ^-1^
${{\rho }}$	Blood and tissue densities	1.06 × 10^3 ^kg m^-3^
${{c}}$^A^	Tissue heat capacity	3.6 × 10^3 ^J (kg K) ^-1^
${{ c}}$^D^	Tissue heat capacity in dead cells	0.67 × 10^3 ^J (kg K) ^-1^
*ω*	Heat transfer coefficient	27.11 × 10^5 ^W (m^3^ K) ^-1^
*T*_*blood*_	Blood temperature	37 °C
*T*_*source*_	Source temperature	105 °C
*A*_*source*_	Ellipsoid source long axis length	27 mm
*B*_*source*_	Ellipsoid source short axis length	22 mm
*D*_*source*_	Ellipsoid source distal throw	0 mm
*K*_*f*_*(T)*	Cell death forward rate coefficient	0.00333 × *e* ^(T/40.5)^ × (1-A) × 10^3^ s^-1^
*K*_*b*_	Cell death backward rate coefficient	0.00777 × 10^3 ^s^-1^

### Clinical evaluation

We evaluated whether the measured characteristics of HCC localization and its environment: distance to the hepatic capsule, type of the closest vessel (portal vein, hepatic vein or hepatic artery), diameter of the closest vessel at its closest point and its distance to the border of the tumor, could be predictive of the ablation volume. We calculated Pearson correlation coefficients between ablation volume and each parameter to examine linear relationships.

The simulated ablation zones were compared against the ground truth, *i.e*., the segmented ablation zones, as illustrated in Fig. 1.

The ablation zones were compared in terms of relative volume difference ${RVD}=|{{{{\rm{V}}}}}_{{{{\rm{gt}}}}}-{{{{\rm{V}}}}}_{{{{\rm{s}}}}}|/{{{{\rm{V}}}}}_{{{{\rm{gt}}}}}$ and Volume Similarity ${VS}=2* ({{{{\rm{V}}}}}_{{{{\rm{gt}}}}}-{{{{\rm{V}}}}}_{{{{\rm{s}}}}})/({{{{\rm{V}}}}}_{{{{\rm{gt}}}}}+{{{{\rm{V}}}}}_{{{{\rm{s}}}}})$ where *V*_s_ is the simulated ablation zone volume and V_gt_, the ground truth volume. To aid interpretation, *RVD* is a unitless relative error, *i.e*., ${RVD}=0.0$ indicates perfect volumetric agreement, and ${RVD}=0.30$ corresponds to a 30% volume difference.

### Statistical analysis

The normality of data distribution was assessed using the Kolmogorov–Smirnov test. For normally distributed parameters, results are presented as mean +/- standard deviation along with the 95% confidence interval (CI) expressed as lower and upper limits. For non-normally distributed parameters, results were reported as median with interquartile range (IQR), defined by the 25^th^ (Q1) and 75^th^ (Q3) percentiles. Comparisons between the two models were performed using either an unpaired Student *t*-test or a Mann–Whitney *U*-test, depending on the data distribution. A *p*-value < 0.05 was considered statistically significant.

## Results

In total, 96 ablations, performed in 65 patients (57 males, 8 females), were investigated in this study. 22 patients had more than 1 tumor ablated. All patients are presented with one or multiple HCC diagnosed by CT, magnetic resonance imaging or biopsy. Patient data, including gender, age, cirrhosis etiology, liver background, alpha foetoprotein level, Eastern Cooperative Oncology Group–ECOG performance status, Barcelona Clinic Liver Cancer–BCLC stage, Child-Pugh class, previous treatments, tumor burden and number of tumors, are reported in Table 2. HCC characteristics, localization, and its environment are reported in Table 3. The median tumor volume and long axis length were 0.85 cm^3^ (IQR: 0.44–1.81) and 14.18 mm (IQR: 11.76–19.95), respectively. The manual processing of a single ablation case required approximately 30 min, encompassing manual annotation of the needle position and manual segmentation of both the tumor and ablation zone.

**Table 2:** Characteristics of the study patients

Variables		Value *N* (%) or median (Q1–Q3)
Gender	Male	57 (87.7%)
	Female	8 (12.3%)
Age at the time of the procedure		62 (57–69) years
Cirrhosis etiology	Alcohol	24 (36.9%)
	HCV	14 (21.5%)
	NASH	6 (9.2%)
	Alcohol + HCV	4 (6.2%)
	HBV	1 (1.5%)
	Miscellaneous	1 (1.5%)
HCC diagnosis	Biopsy	24 (25.0%)
	Imaging	72 (75.0%)
Liver background	F0	1 (1.5%)
	F1	0 (0%)
	F2	1 (1.5%)
	F3	1 (1.5%)
	F4	62 (95.5%)
AFP level	< 10 ng/mL	38 (58.5%)
	≥ 10 ng/mL	27 (41.5%)
ECOG performance status	0	11 (16.9%)
	1	41 (63.1%)
	2	13 (20.0%)
BCLC stage	A	51 (78.5%)
	B	12 (18.5%)
	C	2 (3%)
Child-pugh class	A	59 (90.8%)
	B	5 (7.7%)
	C	1 (1.5%)
Previous treatment	None	33 (50.8%)
	Thermoablation	10 (15.4%)
	TACE	14 (21.5%)
	SIRT	5 (7.7%)
	Resection	3 (4.6%)
	Transplantation	0 (0%)
Tumor burden	Unilobar	54 (83.1%)
	Bilobar	3 (4.6%)
	Solitary	1 (1.5%)
	Multiple	7 (10.8%)
Number of tumors	1	45 (69.2%)
	2	15 (23.1%)
	3	5 (7.7%)

*AFP* Alphafetoprotein, *BCLC* Barcelona Clinic Liver Cancer, *ECOG* Eastern Cooperative Oncology Group, *HCC* Hepatocellular carcinoma

**Table 3:** Overview of the HCC localizations, characteristics, and environment for all tumors (*N* = 96)

Tumor variables		Value *N* (%) or median (Q1–Q3)	[min: max]
Localization	SI	4 (4.2%)	
	SII	11 (11.5%)	
	SIII	13 (13.5%)	
	SIV	15 (15.6%)	
	SV	11 (11.5%)	
	SVI	17 (17.7%)	
	SVII	8 (8.3%)	
	SVIII	17 (17.7%)	
Volume		0.85 cm^3^ (0.44–1.81)	[0.11: 16.43]
Long axis length		14.18 mm(11.76–19.95)	[5.62: 39.63]
Distance to capsule		53.5 mm (34.75–73.25)	[16: 140]
Vessel around	(< 15 mm of distance)		89 (92.71%)
	Type of nearest vessel	Hepatic artery	0 (0.00%)
		Portal vein	56 (58.33%)
		Hepatic vein	33 (34.38%)
	Distance	5.0 mm (0.0–9.0)	[0: 15]
	Diameter	4.0 mm (3.0–5.0)	[3: 16]

*HCC* Hepatocellular carcinoma, *Q1* First quartile, Q3 Third quartile

Ablation durations were available for 87 of 96 procedures (90.6%). The median ablation time was 420 s (IQR: 300–600) (7 min) with an ablation time ranging from 185 s (~3 min) to 2,520 s (42 min). The final temperature recorded at the tip of the needle (directly measured) was available for 78 procedures (81.25%), and the mean final temperature was 80.97 ± 7.36 °C (95% CI: 79.31, 82.63) with final temperatures ranging from 64°C to 95°C. The median ablation volume was 24.07 cm^3^ (IQR: 17.29–27.69), ranging from 7.90 cm^3^ to 78.80 cm^3^. On average, the ablation long axis length was 47.62 ± 8.17 mm (95% CI: 45.97, 49.28). All the ablation information is reported in Table 4.

**Table 4:** Overview of the ablation information and the post-operative ablation zone characteristics for all tumors (*N* = 96)

Ablation Variables	Unit	Number *n* (%)	Mean ± std (95% CI)	Median (Q1–Q3)	[min: max]
Duration	s	87 (90.6%)	-	420 (300–600)	[185: 2520]
Final tip temperature	°C	78 (81.25%)	80.97 ± 7.36 (79.31, 82.63)	82.0 (76.0–86.75)	[64: 95]
Volume	cm^3^	96 (100%)	-	24.07 (17.29–27.69)	[7.90: 78.80]
Long axis length	mm	96 (100%)	47.62 ± 8.17 (45.97, 49.28)	46.60 (42.43– 51.38)	[30.85: 78.58]

*CI* Confidence interval, *Q1* First quartile, *Q3* Third quartile, *SD* Standard deviation

**Fig. 2:**
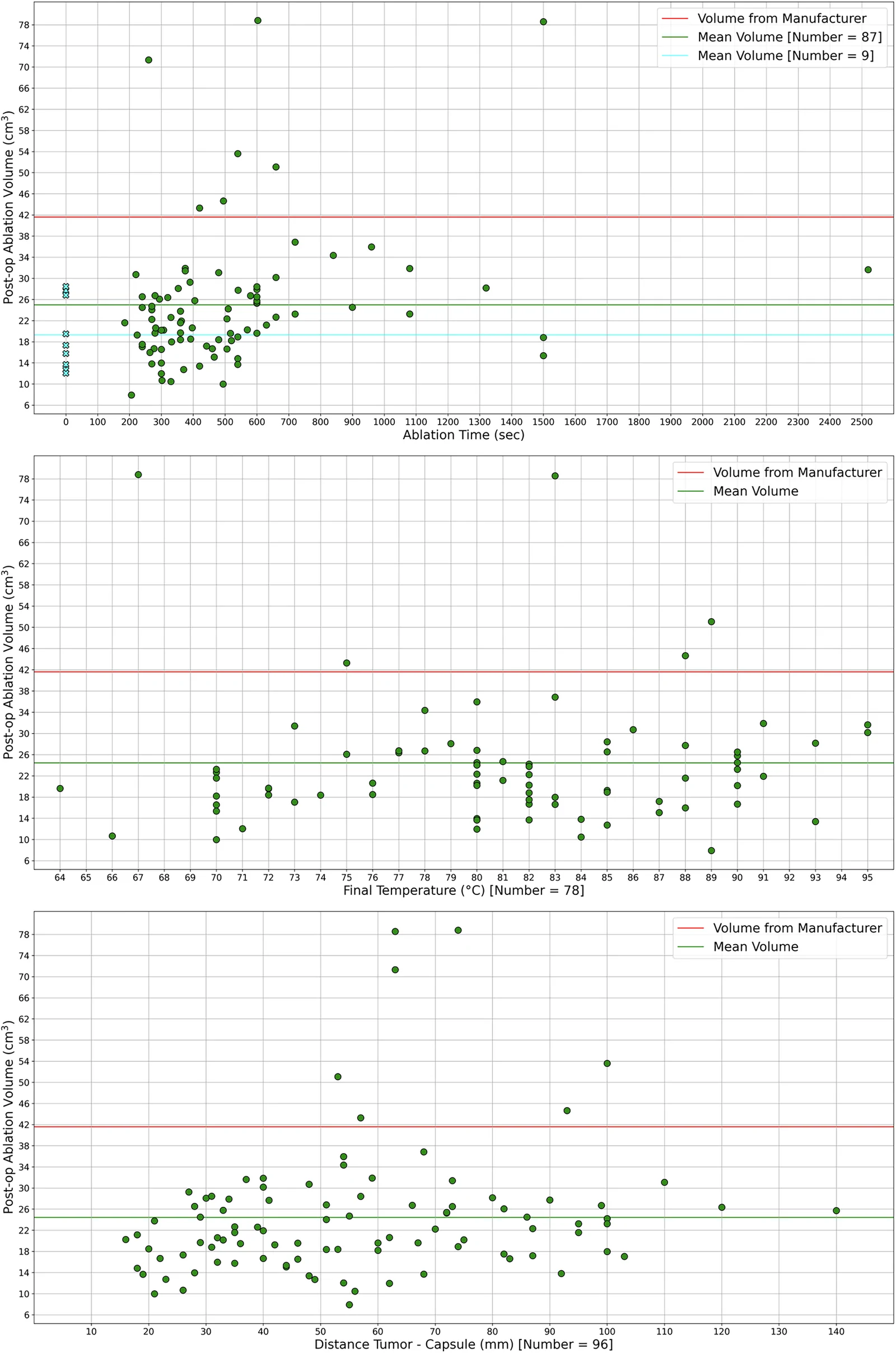
Effect of the ablation duration (top), the final temperature measured at the tip of the probe (middle; measurement was only available for a subset of 78 cases), and the distance between the tumor and the liver capsule on the post-RFA ablation volume. The calculated Pearson correlation coefficients were 0.29, 0.043, and 0.22, respectively, for the ablation time, final temperature, and distance tumor-capsule. RFA, Radiofrequency ablation

**Fig. 3:**
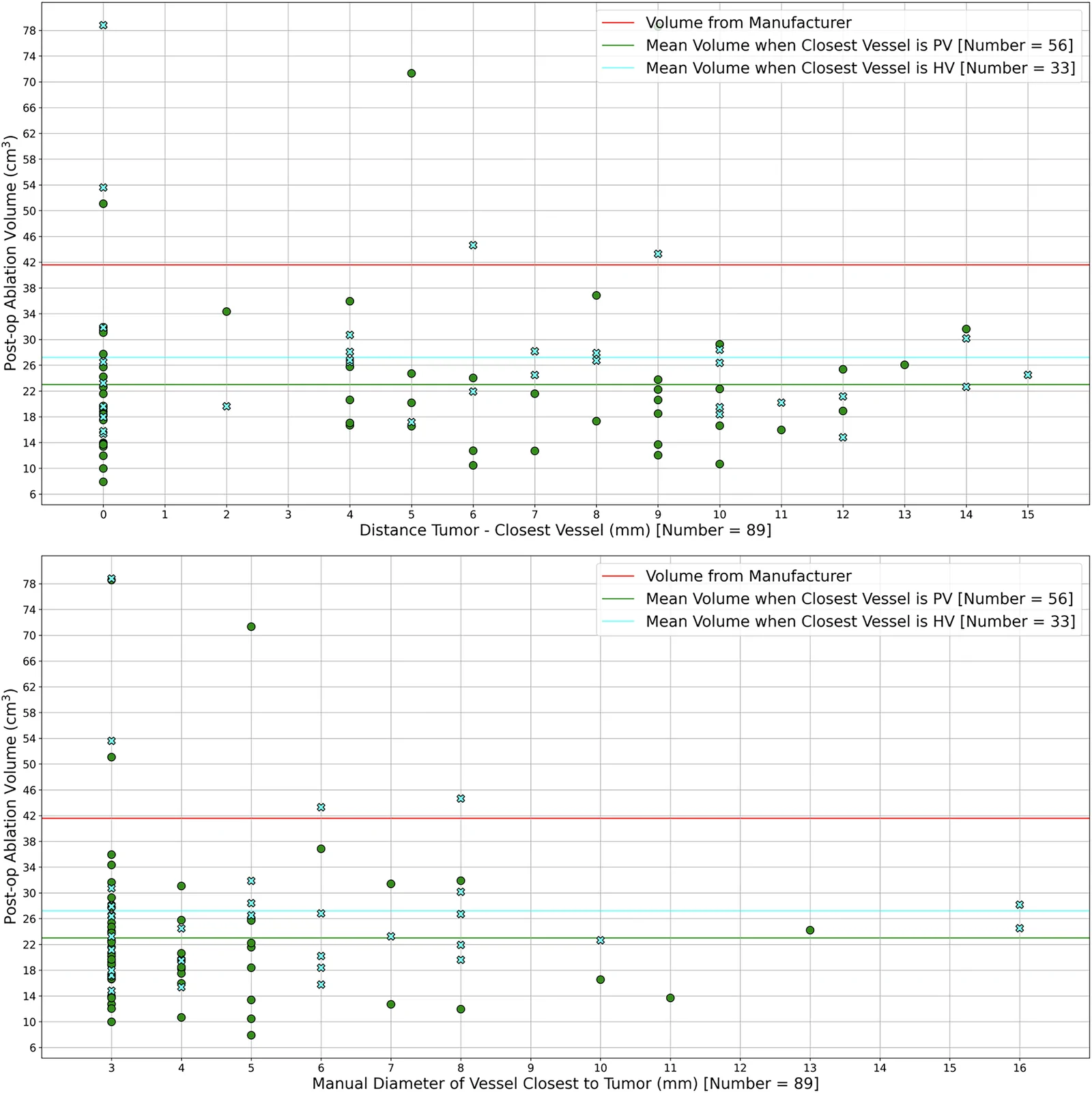
Effect of the distance (top) and diameter (bottom) of the closest vessel to the tumor (*n* = 89). In this case, the dataset was divided into two subgroups: in green, the cases (*n* = 56) for which the closest vessel was a portal vein (PV) and in cyan, the ones (*n* = 33) for which the closest vessel was a hepatic vein (HV). The calculated Pearson correlation coefficients were *r* = 0.0017 and *r* = -0.022 for the distance and diameter, respectively

As illustrated in Fig. S1, even for a standardized ablation protocol and a similar patient cohort, the final ablation volume showed a wide range, 7.9 to 78.8 cm^3^ across the entire dataset. Therefore, we first investigated whether we could identify parameters that influenced the final volume of the ablation zone. As shown in Fig. 2 and Fig. 3, none of those parameters showed a statistically significant correlation with the final ablation volume (all *r* = -0.022 to 0.29).

To ensure a fair comparison with the manufacturer’s estimate, we calibrated A_sourc__e_, B_source_, and D_source_ to ensure that the model produced the exact same ablation zone when a needle was placed at the center of the computational domain without any blood vessels, simulating an *ex vivo* experiment. The calibrated values are reported in Table 1. In this case, the ablation zone was an ellipsoid with semiaxis *A* = 45 mm and *B* = 42 mm, corresponding to the ablation zone estimated by the manufacturer.

We simulated the ablation zones using anatomical information extracted from the pre-RFA CT scan and needle information gathered from the intra-procedure CT scan, and compared them with the patient-specific ground truth from the post-RFA images to evaluate the model predictions. The mean computational time to simulate 1 s of ablation was 0.45 ± 0.13 s (95% CI: 0.42, 0.47) on a NVIDIA RTX A4500 GPU.

An automated segmentation algorithm was run on the 96 ablations, but two cases were excluded from the model evaluation due to poor liver segmentation quality. One case presented a major right hepatectomy, and the other had a lipiodol injection. The limited performance can be attributed to the absence of these scenarios in the deep learning algorithm's training data.

Ablation times were available for 85 of 94 procedures (90.4%). For the nine procedures for which the ablation time was unknown, we assumed an ablation time of 12 min, as prescribed by the manufacturer.

Simulation results are presented in Table 5. Across the entire dataset, the simulated and segmented ablation zone volumes were similar (*p* = 0.165), while the difference between the manufacturer estimation and segmented ablation zone volumes was significantly different (*p* < 0.001). The model achieves a median RVD of 0.39 (IQR: 0.24–0.57), representing a significant improvement (*p* < 0.001) over the manufacturer’s estimate with a median RVD of 1.04 (IQR: 0.64–1.61).

**Table 5:** Comparison of ablation volumes from clinical measurements, biophysical simulation, and manufacturer estimates, with relative volume difference (RVD) between simulation and manufacturer values and the post-RFA ground truth, reported for the overall cohort and stratified by cases with known *versus* unknown ablation time

Ablation variables	Unit		Median (Q1, Q3)	[min: max]	*t*-test *p*-value
All results (*N* = 94)					
Volume	cm^3^	Post-RFA	22.07 (17.22, 27.45)	[7.9: 78.8]	
		Simulation	23.82 (15.96, 36.06)	[11.8: 324.69]	0.16
		Manufacturer	45.02 (44.96, 45.09)	[43.21: 46.65]	< 0.0001
RVD	-	Simulation	0.39 (0.24, 0.57)	[2.28e^-02^: 9.27]	
		Manufacturer	1.04 (0.64, 1.61)	[0.01: 4.69]	< 0.0001
Results with personalized ablation time (*N* = 85)					
Volume	cm^3^	Post-RFA	22.33 (17.97, 27.74)	[7.9: 78.8]	
		Simulation	21.39 (15.76, 30.65)	[11.8: 324.69]	0.93
		Manufacturer	45.02 (44.97, 45.09)	[43.21, 46.65]	< 0.0001
RVD	-	Simulation	0.36 (0.22, 0.52)	[2.28e^-02^: 9.27]	
		Manufacturer	1.02 (0.63, 1.48)	[0.01: 4.69]	< 0.0001
Results with standard ablation time (*N* = 9)					
Volume	cm^3^	Post-RFA	17.33 (13.68, 26.81)	[12.04: 28.46]	
		Simulation	40.83 (38.23, 43.31)	[31.91: 46.04]	< 0.0001
		Manufacturer	45.01 (44.87, 45.06)	[43.47: 45.12]	< 0.0001
RVD	-	Simulation	1.33 (0.52, 1.36)	[0.38: 2.74]	
		Manufacturer	1.60 (0.68, 2.29)	[0.57: 2.74]	0.44

*Q1* First quartile, *Q3* Third quartile, *RFA* Radiofrequency ablation, *RVD* Relative volume difference, *SD* Standard deviation

**Fig. 4:**
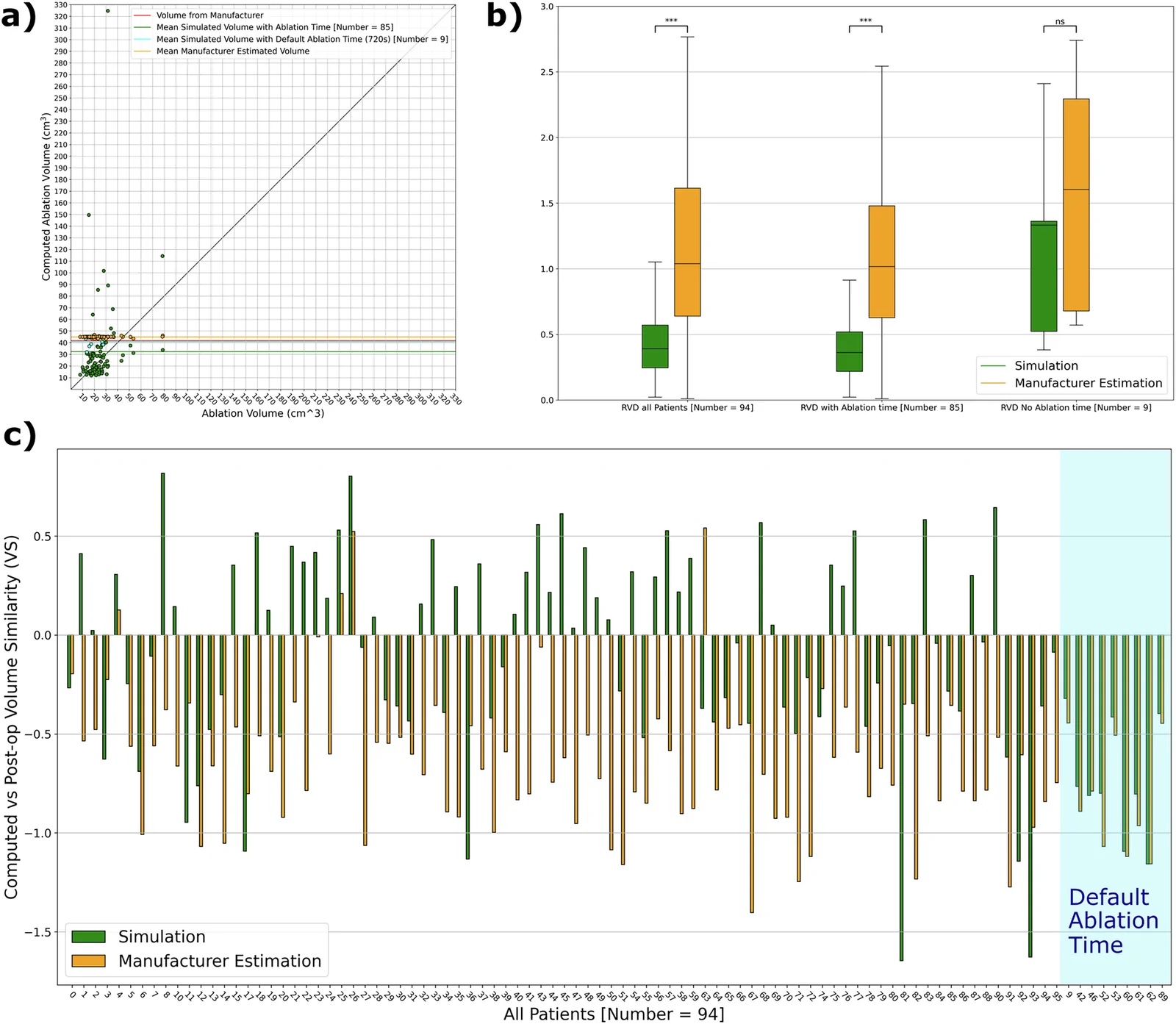
**a** Comparison between the ablation volume observed on the post-RFA CT and computed ablation zone using the proposed model (green: patients with ablation time, blue: patients with standard ablation time) or using the estimation from the manufacturer (yellow). **b** Relative volume difference (RVD) computed between the ground truth segmented ablation zone and the ablation zone estimated with the proposed model (green) or with the manufacturer's estimation (yellow). Results are shown considering the entire dataset and separately for patients where the ablation time was known or not. **c** Volume similarity (VS) between the computed and the post-RFA ablation zones across the entire patient cohort. A positive VS corresponds to an underestimation of the actual ablation zone, while a negative VS corresponds to an overestimation. CT, Computed tomography; RFA, Radiofrequency ablation

Figure 4 shows scatter plots of the model-predicted ablation volume and the manufacturer’s estimation compared to the ground truth post-RFA volume (top left). As expected, the manufacturer’s estimated volumes remain constant in all cases while the model predictions are closer to the ground truth, consistent with the significantly lower RVD compared to the manufacturer’s estimate (*p* < 0.001). Boxplots extending from Q1 to Q3, with a line at the median, are shown in Fig. 4 (top right) with results for the full patient cohort (*n* = 94), for patients with known ablation times (*N* = 85) and for those for whom the standard ablation time was applied (*n* = 9).

We also computed the volume similarity to analyze if a constant overestimation and/or underestimation of the ablation zone was present in the model and the manufacturer's estimation. Figure 4 (bottom) shows the results for the entire dataset. No systematic over-ablation or under-ablation was observed based on similarity analysis, except when the ablation time was not known, in this case, both the model and the manufacturer overestimated the post-RFA ablation volume.

**Fig. 5:**
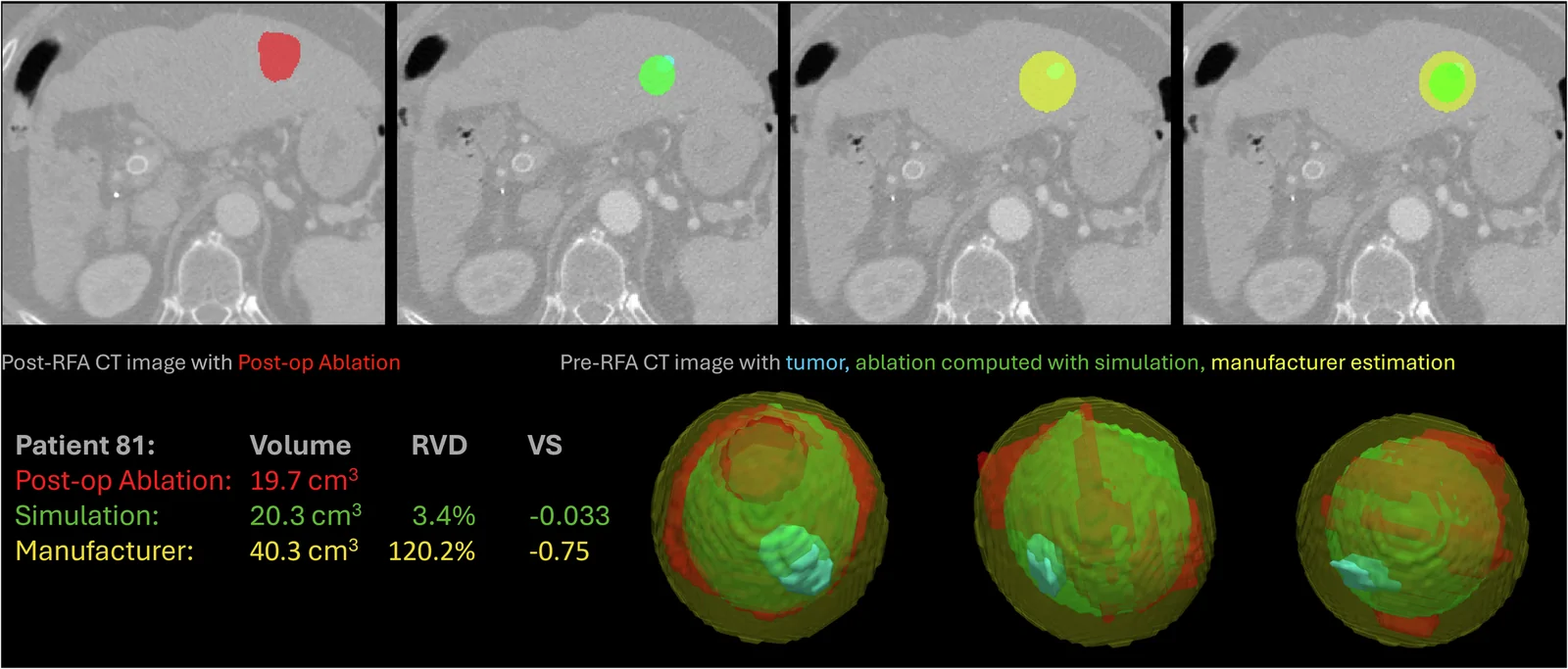
Prediction results for patient #81. (Top) Ablation zone predicted with the proposed model (green) aligned qualitatively well with the ground truth (red) and performed better than the manufacturer's estimation (yellow). The tumor is shown in cyan on the pre-RFA CT image. (Bottom) 3D rendering of the tumor (cyan) and the ablation zones segmented from post-RFA CT image (red), predicted with the proposed model (green) and predicted by the manufacturer (yellow). In the three-dimensional rendering, the post-RFA ablation zone was manually transposed to enable a visual comparison among the three ablation volumes. CT, Computed tomography; RFA, Radiofrequency ablation

Figure 5 (right) illustrates how the ablation zone predicted by the biophysics-based model visually aligned with the ground truth (illustrative example) for patient 81.

## Discussion

We evaluated a fully automated, artificial intelligence-powered, patient-specific biophysical model of RFA on 94 HCC lesions and showed that the model was fast: 1 s of ablation computed in 0.45 ± 0.13 s (95% CI: 0.42, 0.47) and significantly more accurate than the manufacturer's approximation at estimating the ablation volume: median model RVD: 0.39 (IQR: 0.24–0.57) *versus* median manufacturer RVD: 1.04 (IQR: 0.64–1.61). As we rely on a deep learning segmentation method, our pipeline is fully automated, enabling scalable and reliable evaluation by eliminating manual steps prone to human error. It also allows direct analysis of intra-procedure data, rather than relying on diagnostic images, allowing seamless integration into clinical practice without the need for complex image registration.

To the best of our knowledge, this is the first report focused on HCC with a patient cohort reflecting real-world clinical conditions. Indeed, the included patients had underlying liver fibrosis or cirrhosis, which results in heterogeneous liver parenchyma. This is significantly different from the healthy, homogeneous and not perfused liver tissue typically used in *ex vivo* studies by device manufacturers to define standard ablation volumes. While the current model still relies on nominal parenchymal tissue properties rather than fibrosis- or cirrhosis-specific values, it nonetheless yielded substantially more accurate volume predictions compared to the manufacturer reference (median RVD 0.39 *versus* 1.04), demonstrating a clear benefit even in this heterogeneous patient population. We expect that explicitly incorporating fibrosis- and cirrhosis-dependent tissue properties into our biophysical model will further improve prediction accuracy, a focus of our future work.

We analyzed several parameters that had the potential to influence the ablation volume, such as the ablation time, final recorded temperature, distance between the tumor and liver capsule and the distance, type and diameter of the closest vessels. None of these parameters predicted the ablation zone, nor could they explain the large variability observed in the resulting ablation volumes. In contrast, the model integrates all those factors and explicitly accounts for the heat-sink effect of the large blood vessels, an aspect ignored by the manufacturer's estimate. This comprehensive approach enables the model to achieve promising predictions of ablation volume that show a statistically significant correlation with the post-RFA ablation zones.

Another key takeaway is the importance of incorporating personalized procedural data, such as the actual ablation time, which led to more accurate predictions than using the manufacturer’s standard ablation duration. In our center, we do not use the 12-min standard ablation duration. Instead, we wait to obtain 3 roll-offs, regardless of the ablation time. The total ablation time is documented in the ablation report, which allowed us to use this parameter in the current analysis. However, the precise duration and timing of each of the 3 roll-offs is not reported and therefore cannot be considered. Integrating this information for each patient would likely enhance the accuracy of our results. Another limitation of our study is the use of nominal tissue parameters, which do not reflect inter-patient variability in hepatic perfusion or tissue heterogeneity.

In our study, we focused on predicting the ablation volume, rather than its spatial extent. Evaluating the spatial distribution requires an elastic registration between the pre-procedure CT images used for simulation and the post-procedure CT images in which the ablation zone is segmented, without unrealistically distorting the tumor area. Currently, no robust, fully automatic registration algorithms tailored to ablation data are available. As a result, such an approach would require manual registration or the exclusion of cases in which registration fails. Future work will focus on integrating robust automated deformable image registration techniques to enable voxel-wise comparison between predicted and observed ablation zones. Such approaches would allow a more detailed assessment of the spatial distribution of necrosis.

## Conclusion

Developing predictive models for RFA remains a complex task, especially in heterogenous liver backgrounds such as cirrhosis. By systematically integrating patient-specific anatomy and procedural data, computational models hold great potential to standardize the planning process, enhance consistency, reduce reliance on operator expertise, and improve clinical outcomes, ultimately democratizing access to effective thermal ablation. A key next step will be to evaluate whether model performance correlates with clinically meaningful outcomes, such as local tumor progression, recurrence-free survival, and ablative margin adequacy. The present study focuses on establishing a robust and validated biophysical model as a necessary foundation for clinical translation. The intended next step is to deploy this model in a preoperative planning setting, using pre-procedural imaging as input, which would allow the model to recommend an optimal ablation strategy, including the number of needles and their positioning, with the goal of achieving complete tumor coverage while minimizing damage to surrounding healthy tissue.

## Supplementary information

**Additional file 1: Fig. S1** Ablation volume across the entire dataset (*n* = 96).

## Data Availability

The data is available upon reasonable request to the corresponding author.

## Abbreviations

CI: Confidence interval
GPU: Graphics processing unit
HCC: Hepatocellular carcinoma
IQR: Interquartile range
RFA: Radiofrequency ablation
RVD: Relative volume difference
