# Supplementary material for: Patient-specific biophysical AI-powered modeling for predicting RFA volumes in HCC
Source: Eur Radiol Exp. 2026 Nov 3;10:166. doi: 10.1186/s41747-026-00839-3 (PMC13731545; doi:10.1186/s41747-026-00839-3)
Supplement: Supplementary file 1 [file 41747_2026_839_MOESM1_ESM.pdf]

Patient-specific biophysical AI-powered modeling for predicting RFA volumes in HCC

Electronic Supplementary Material

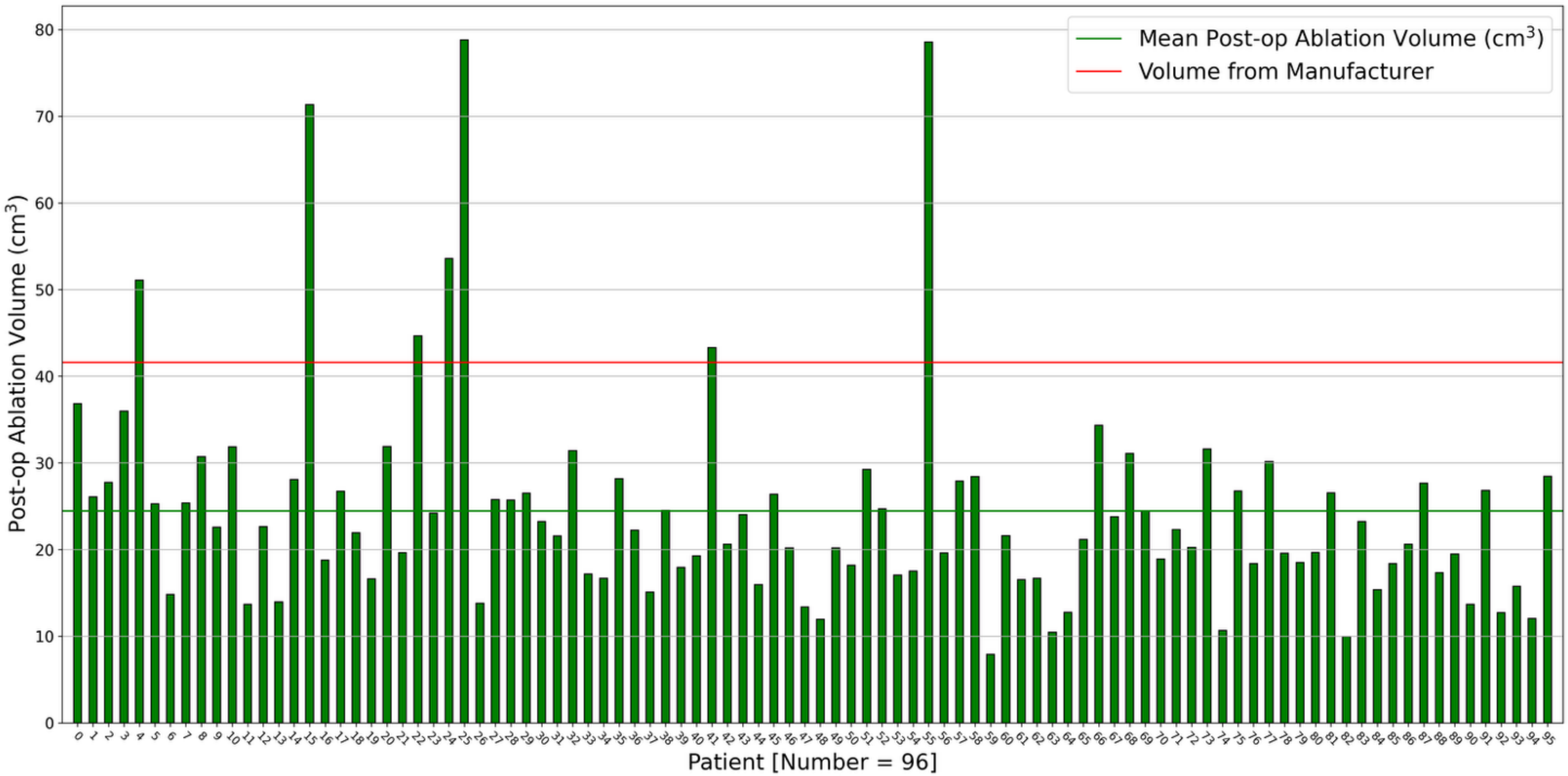

Fig. S1 Ablation volume across the entire dataset (*n* = 96)
